# Investigating the roles of academic emotions in the relationship between agentic engagement and EFL achievement among Chinese undergraduate students

**DOI:** 10.3389/fpsyg.2025.1661196

**Published:** 2025-12-17

**Authors:** Hongwu He

**Affiliations:** School of Education, Shanghai International Studies University, Shanghai, China

**Keywords:** academic emotions, agentic engagement, EFL achievement, grade level comparison, Chinese undergraduate students

## Abstract

This study examines the relationships among agentic engagement, academic emotions, and English as a foreign language (EFL) achievement in Chinese undergraduate students, with a focus on variations across grade levels. Utilizing a quantitative design, data were collected from 221 English majors (111 first-year and 110 fourth-year undergraduate students) via questionnaires assessing agentic engagement and academic emotions, alongside English examination scores. Data analysis was conducted using SPSS Amos to explore moderating and mediating effects. Results reveal that none of the academic emotions showed significant moderating effects. Pride stands out because it fully mediated the impact of agentic engagement on English achievement across the entire sample (indirect effect = 0.246, *p* < 0.05), while hopelessness and anxiety demonstrated partial mediating effects. Grade-level differences were stark: for first-year undergraduate students, agentic engagement directly boosted EFL achievement, with emotions playing a negligible role. Conversely, among fourth-year undergraduate students, positive emotions fully mediated this relationship (indirect effect = 0.174, *p* < 0.05), with no significant direct effect. These findings highlight the stage-specific roles of academic emotions, with hopelessness amplifying engagement’s effect broadly and positive emotions like pride driving achievement in later years. Theoretically, this enriches models of engagement and emotion in EFL learning, emphasizing cultural and developmental nuances in China’s educational context. Practically, it suggests tailored strategies—promoting proactive engagement for novices and fostering positive emotions for advanced learners—to enhance EFL outcomes.

## 1 Introduction

As English as a foreign language (EFL) learning takes center stage in China’s higher education system, understanding the psychological mechanisms that underlie achievement is paramount. Among these mechanisms, students’ proactive involvement in their learning process—known as agentic engagement—and the emotions they experience in academic settings stand out as critical yet underexplored factors. This study investigates the associations among these elements and the potential role of emotions in the engagement-achievement link, offering fresh insights from a culturally distinct educational context.

Agentic engagement reflects students’ intentional efforts to shape their learning, such as setting goals, seeking feedback, and overcoming obstacles (Reeve and Tseng, 2011). Research has shown that such proactive behaviors enhance academic outcomes by fostering autonomy and persistence (Zimmerman, 2002). Recent investigations among Chinese university EFL students further affirm that agentic engagement, mediated by mastery-approach goals, enhances persistence and performance in language tasks (Zhong and Zhan, 2024). Similarly, academic emotions—ranging from anxiety and boredom to enjoyment and pride—play a pivotal role in modulating learning processes (Pekrun et al., 2002). Positive emotions can amplify motivation and focus, while negative emotions may impede progress, particularly in language learning where emotional responses are often intensified by performance pressure (Pekrun, 2006). In Chinese EFL settings, studies reveal that foreign language enjoyment positively correlates with self-perceived proficiency, while classroom anxiety inversely predicts it, underscoring emotions’ role in modulating cognitive and behavioral investment (Wu, 2024).

Yet, the dynamic between agentic engagement and these discrete emotions, particularly in relation to EFL achievement, lacks comprehensive exploration, especially among Chinese learners where cultural emphases on collectivism and academic conformity may distinctly modulate these links (Zhong and Zhan, 2024).

This study is significant for examining how specific positive and negative emotions interact with agentic engagement to influence EFL achievement, particularly across university grade levels, where Chinese undergraduate students’ emotional responses and engagement strategies evolve from novice (first-year) to advanced (fourth-year) stages (Liu, 2006). Although prior research has separately linked engagement and emotions to outcomes (Shao et al., 2020), their combined effects remain underexplored in non-Western, exam-oriented EFL contexts. By bridging theoretical insights into emotion-engagement dynamics with practical strategies to harness positive emotions and mitigate negative ones, this research illuminates affective-behavioral pathways to EFL success, informing tailored pedagogical support for Chinese undergraduates.

## 2 Literature review

The pursuit of English proficiency has become a defining feature of higher education in China, where it serves as a gateway to academic advancement and global opportunities (Hu, 2005). Within this context, understanding the psychological factors that underpin EFL achievement is essential, particularly as students navigate the emotional and behavioral complexities of language learning. Over the past decade, researchers have increasingly turned their attention to the roles of student engagement and academic emotions in shaping learning outcomes. However, the specific interplay between agentic engagement—a proactive form of student involvement—and the diverse array of academic emotions in influencing EFL achievement remains a largely uncharted territory, especially among Chinese undergraduate students. This literature review synthesizes existing research on these constructs, explores their relationships with EFL achievement, and highlights critical gaps that necessitate further investigation, particularly within the culturally unique Chinese educational landscape.

### 2.1 Agentic engagement

Agentic engagement, introduced by Reeve and Tseng (2011), refers to students’ intentional efforts to enhance their learning experiences, such as by setting personal goals, seeking constructive feedback, and managing challenges. Unlike traditional dimensions of engagement—behavioral, emotional, and cognitive—agentic engagement emphasizes students’ agency, positioning them as active contributors to their educational process. Studies have demonstrated its positive impact on academic achievement across various domains. For instance, Reeve (2013) found that agentic engagement predicted higher grades and increased motivation among Korean high school students, a finding that resonates with East Asian educational contexts where self-directed learning can complement teacher-centered approaches. In the realm of EFL learning, where persistence and active participation are vital, agentic engagement holds particular promise. Mercer (2019) has argued that engaged language learners exhibit greater willingness to communicate and overcome linguistic barriers, yet her work focuses broadly on engagement rather than the specific agentic dimension. While Hiver et al. (2020) explored proactive behaviors in language classrooms, their study does not explicitly link agentic engagement to EFL achievement, leaving a gap in understanding its direct effects, especially among Chinese learners where cultural norms may shape its expression (Huang et al., 2022). Recent research in Chinese EFL contexts has begun to address these gaps. Jiang and Zhang (2021) identified teacher autonomy support and social relatedness as significant predictors of agentic engagement, mediated by mastery-approach goals—highlighting its relevance in exam-oriented environments. Similarly, Zhong et al. (2025) found that project-based learning fosters agentic engagement in speaking activities, though cultural preferences for subtle proactivity may moderate its effects. Collectively, these findings underscore the potential of agentic engagement to enhance EFL learning outcomes, yet they also point to a critical research need: a deeper understanding of the discrete emotions that prompt or sustain such proactive learner behaviors.

### 2.2 Academic emotions

Parallel to engagement, academic emotions have emerged as a critical lens through which to view learning processes. Defined as emotions tied to academic activities and outcomes, these include both positive feelings like enjoyment, pride, and hope, and negative ones such as anxiety, boredom, and shame (Pekrun et al., 2002). Pekrun’s (2006) control-value theory provides a robust framework, suggesting that emotions arise from students’ appraisals of control and value in learning tasks, influencing motivation, cognition, and performance. In EFL contexts, emotions take on heightened significance due to the personal and social stakes of language proficiency. Language anxiety, one of the most studied emotions, has been consistently linked to reduced performance and communication apprehension (Horwitz et al., 1986; MacIntyre and Gardner, 1994). Conversely, Dewaele and MacIntyre (2014) found that foreign language enjoyment enhances motivation and outcomes among multilingual learners, underscoring the dual role of emotions in facilitating or hindering success. Within China, where English learning is often tied to high-stakes assessments like the College English Test, negative emotions such as anxiety are prevalent (Liu, 2006), yet positive emotions like hope or pride remain underexplored. Shao et al. (2020) emphasized the need for a broader examination of achievement emotions in language learning, but their review stops short of linking these to specific engagement types. Furthermore, latent profile analyses of pre-exam emotions among Chinese high school EFL students identify distinct profiles (e.g., high anxiety-low enjoyment) that predict achievement variability, with positive emotions like pride buffering against boredom (Shi and Wang, 2025). These insights affirm emotions’ domain-specificity in EFL but underscore the need for emotion-engagement integrations in Chinese contexts.

Although foreign language anxiety (Horwitz et al., 1986) and foreign language enjoyment (Dewaele and MacIntyre, 2014) dominate the emotion–performance literature, a growing body of evidence highlights the distinct predictive power of other discrete emotions. Pekrun et al. (2017) demonstrated in a large-scale longitudinal study that pride, hope, anger, shame, and boredom each exert unique effects on academic achievement, even after controlling for anxiety and enjoyment. This creates a conceptual tension: treating emotions as broad valence-based constructs risks masking the differentiated pathways through which specific emotions shape motivation, strategy use, and performance (Pekrun and Perry, 2014). A broader palette of discrete emotions may enable a more nuanced understanding of how particular emotional experiences interact with proactive engagement behaviors.

### 2.3 Interplay of agentic engagement and academic emotions in EFL learning

Understanding the precise mechanisms through which agentic engagement influences EFL achievement remains a central challenge. The broader reciprocal relationship between academic emotions and engagement is well-established in general educational literature (e.g., Skinner et al., 2008; Linnenbrink-Garcia et al., 2011). Within EFL research, recent studies such as that by Wang et al. (2023) demonstrate that engagement acts as a mediator between broader emotional factors like Foreign Language Enjoyment and Anxiety and eventual achievement, which effectively positions engagement as a key outcome of emotions. However, an alternative perspective, grounded in control-value theory (Pekrun, 2006) suggests that emotions themselves may function as a central mediator between learning antecedents and outcomes. The specific question of how academic emotions might transmute the effects of proactive participation into success or failure is therefore left unanswered, a lacuna that is particularly salient given the high-stakes nature of many EFL learning environments.

The Chinese educational context adds a layer of complexity to these relationships. Characterized by an emphasis on examination success and respect for authority, Chinese classrooms shape how students engage and experience emotions (Hu, 2005). English proficiency carries significant weight, often determining academic and career trajectories, which can intensify emotional responses like anxiety or hope (Liu, 2006). Cultural values, such as collectivism, may also influence agentic engagement, with students opting for subtle forms of proactivity, such as private feedback-seeking, rather than overt participation (Huang et al., 2022). While some studies have examined engagement or emotions among Chinese EFL learners, few integrate these constructs. For example, Liu (2006) focused on anxiety’s impact on performance, but did not explore its interaction with engagement strategies. Similarly, Reeve’s (2013) findings in Korea suggest agentic engagement’s relevance in East Asia, yet its application to Chinese undergraduate students remains untested.

### 2.4 Research gap and research questions

Despite these advances, several critical shortcomings persist in the literature. First, while agentic engagement and academic emotions have been linked to academic outcomes independently, their combined influence on EFL achievement is rarely examined, particularly in non-Western settings. Existing research often treats emotion as a broad variable rather than discrete categories, obscuring the unique roles of specific emotions (Pekrun et al., 2011). Second, the majority of studies are situated in Western contexts, limiting their applicability to China, where cultural and educational factors may alter these dynamics (Bond, 1991; Tao and Hong, 2014). For instance, negative emotions in Western contexts often leads to disengagement, yet in collectivist settings it may be suppressed and redirected into heightened persistence to restore group harmony and personal honor (Markus and Kitayama, 2010). Third, cross-sectional designs predominate and little attention has been paid to how these relationships evolve across different stages of undergraduate education. Emotional regulation and agentic behaviors are not static; they develop across the undergraduate trajectory. First-year undergraduate students typically experience higher transition-related anxiety and lower perceived control (Wang and Eccles, 2013), whereas fourth-year undergraduate students possess greater metacognitive awareness, emotional regulation skills, and language proficiency—potentially altering which emotions most strongly facilitate or impede agentic engagement (Shirvan et al., 2022). This developmental perspective is largely absent in EFL research, despite its potential to inform stage-specific interventions.

Academic emotions do not operate in isolation but are intrinsically linked to engagement in the learning process. Accordingly, this study examines their potential role in the association between agentic engagement and EFL outcomes. By focusing on how discrete emotions moderate or mediate the relationship between agentic engagement and EFL achievement, rather than examining them as standalone predictors, this study aims to provide a more nuanced understanding of their interplay. Furthermore, it explores potential variations in these associations across grade levels among Chinese undergraduate students. Accordingly, this study proposes the following research questions:

1. What is the current situation of agentic engagement and discrete academic emotions among Chinese undergraduate students?
2. To what extent do discrete academic emotions mediate or moderate the relationship between agentic engagement and EFL achievement in Chinese tertiary education contexts?
3. How do the moderating and mediating effects of academic emotions on the agentic engagement–EFL achievement link vary across grade levels in Chinese tertiary education?

The study seeks to extend theoretical models of engagement and emotion while offering practical insights for educators to foster effective learning strategies tailored to students’ emotional and developmental needs. In doing so, it responds to the pressing demand for research that bridges psychological processes and EFL achievement in a culturally relevant context, paving the way for enhanced educational practices in Chinese higher education.

## 3 Research methods

This study adopts a quantitative research design to investigate the roles of academic emotions in shaping the relationship between agentic engagement and EFL achievement among Chinese undergraduate students, with a particular emphasis on differences between first-year and fourth-year undergraduate students. By employing a cross-sectional approach, the research seeks to examine how discrete academic emotions mediate and moderate the influence of proactive engagement on English achievement, using statistical tools suited for such analyses. This section details the participants, measures, data collection procedures, and data analysis plan, providing a comprehensive framework for exploring these psychological and academic dynamics.

### 3.1 Participants

The participants consist of 221 English major students from a language university in China, including 111 first-year undergraduate students and 110 fourth-year undergraduate students. This sample enables a comparative analysis between new learners at college, who are adjusting to university-level language demands, and advanced learners with greater academic experience and proficiency. English majors were chosen due to their intensive focus on EFL development, which aligns with the study’s objectives. The sample size exceeds the threshold recommended for robust regression-based analyses, ensuring adequate statistical power to detect mediating and moderating effects (Hayes, 2017). Recruitment occurred within intact classes, preserving the naturalistic classroom environment typical of Chinese higher education and enhancing ecological validity.

### 3.2 Instruments

Agentic engagement is assessed with the Foreign Language Learning Agentic Engagement Scale (FLL-AES), developed by Guo and Li (2018). The FLL-AES is a psychometrically validated instrument designed to measure proactive student contributions in foreign language learning contexts. Grounded in Reeve’s (2013) theory of agentic engagement, this scale is based on four dimensions: Self-Directed Learning Effort (autonomous strategies for overcoming challenges), Assisting Teachers (providing resources/feedback), Cooperating with Teachers (active task participation), and Peer Support (sharing knowledge and encouragement). The scale comprises 14 items rated with a 5-point Likert scale (See Appendix A). Psychometric validation demonstrated robust reliability (Cronbach’s *α* = 0.907 overall; subscales *α* = 0.827–0.845) and validity. Exploratory Factor Analysis confirmed the four-factor structure (71.027% cumulative variance explained), while Confirmatory Factor Analysis indicated excellent model fit (CFI = 0.991, RMSEA = 0.035).

Academic emotions are measured using the General Academic Emotion Questionnaire for College Students (GAEQ) by Ma (2008). Grounded in Pekrun’s theoretical framework of academic emotions (2002), the GAEQ comprises 10 subscales measuring these specific emotions: Anxiety, Boredom, Relief, Hopelessness, Pride, Shame, Enjoyment, Hope, Anger, and Interest. Crucially, factor analyses supported a four-dimensional structure aligning with Pekrun’s taxonomy (2002): Negative Activating Emotions (shame, anxiety, anger), Positive Activating Emotions (interest, enjoyment, hope), Negative Deactivating Emotions (hopelessness, boredom), and Positive Deactivating Emotions (pride, relief). This instrument employs a 5-point Likert scale for scoring the 88 items spread in the 10 emotion subclasses (See Appendix B). Psychometric evaluations demonstrated acceptable reliability for the subscales. Internal consistency, measured by Cronbach’s Alpha coefficients, ranged from 0.641 to 0.887, while test–retest reliability over a 4-week interval yielded coefficients between 0.563 and 0.866, indicating reasonable stability. Initial construct validity evidence was gathered through exploratory factor analysis, confirming the hypothesized four-factor model and accounting for 82.202% of the variance.

EFL achievement was assessed in students’ final English examinations and the items were drawn from a common item bank. The items in the item bank covered the domains of grammar, vocabulary, and reading comprehension. The items of each domain were calibrated using the Rasch model and equated based on common anchor items, with raw scores subsequently converted into ability measures of a certain domain. For each student, the mean of ability scores of the three domains—after being linearly transformed to a 100-point scale—was taken as his/her EFL achievement score.

### 3.3 Data collection

Data collection is conducted in two phases to balance self-reported and objective data. The agentic engagement and academic emotions questionnaires, in the original Chinese version, are administered during a scheduled classroom session, supervised by trained research assistants who provide standardized instructions in Chinese to ensure comprehension. This process, lasting approximately 20–30 min, occurs under controlled conditions to minimize external influences and enhance response accuracy. Subsequently, final English examination scores are retrieved from the university’s academic records with institutional approval, ensuring precision and eliminating self-report bias. This dual-method approach integrates psychological insights with tangible academic outcomes, strengthening the study’s evidential base.

Ethical considerations are meticulously addressed to protect participant rights and data integrity. The study is approved by the university’s institutional review board, adhering to ethical research standards. Participants provide informed consent after receiving a clear explanation of the study’s purpose, procedures, and their right to withdraw at any time without consequences. Access to examination scores is restricted to authorized researchers, ensuring confidentiality throughout the process.

### 3.4 Data analysis

The data analysis is conducted using SPSS Amos Version 26. The analysis unfolds in several stages to comprehensively address the research objectives. Initially, preliminary analyses establish the dataset’s foundation. Descriptive statistics—means, standard deviations, skewness, kurtosis, minimum, and maximum values—are calculated to summarize agentic engagement, academic emotions, and EFL achievement for the full sample and by grade level.

To investigate the roles of discrete academic emotions in the relationship between agentic engagement and EFL achievement, the research first conducted moderation analyses for specific academic emotions across the entire sample using Amos. For each emotion, agentic engagement was designated as the independent variable, EFL achievement as the dependent variable, and the specific emotion as the moderator. Interaction terms were created by multiplying mean-centered variables to mitigate multicollinearity, and their significance was assessed using *p*-values to determine whether the emotion moderated the engagement-achievement relationship (Aiken and West, 1991).

Following this, the researcher performed mediation analyses for specific academic emotions across the entire sample using Amos. In these analyses, agentic engagement served as the independent variable, EFL achievement as the dependent variable, and each specific emotion as the mediator. Indirect effects were estimated through bootstrapping with 5,000 resamples, with significance determined by examining whether the 95% Bootstrap confidence intervals excluded zero, as recommended by Preacher and Hayes (2008). The two-tailed *p*-values of indirect effects were determined by whether the bias-corrected confidence interval excluded zero.

To address the increased risk of Type I error arising from multiple comparisons in both moderation and mediation analyses, *p*-values generated in these analyses were adjusted using the Benjamini-Hochberg (BH) procedure (Benjamini and Hochberg, 1995), which effectively controls the false discovery rate (FDR). The BH method offers superior statistical power compared to more conservative corrections such as the Bonferroni adjustment while still successfully managing the proportion of false positives among statistically significant findings. The Benjamini-Hochberg procedure operates by first sorting the original *p*-values in ascending order: $p_{\left(1\right)}\leq p_{\left(2\right)}\leq \cdots \leq p_{\left(m\right)}$ , where *m* represents the total number of hypothesis tests conducted. The corrected *p*-values were calculated with the following formula:


$$p_{\left(k\right)}\leq \frac{k}{m}\cdot q.$$

Here, *m* is the number of tests, *k* is the rank order of the *p*-value in the sorted list and *q* is the desired significance level (e.g., 0.05). Next, the research explored the moderating effects of composite positive and negative emotions on the relationship between agentic engagement and EFL achievement among first-year undergraduate students. Negative emotions, including anxiety, boredom, hopelessness, shame, and anger, and positive emotions, encompassing relief, pride, enjoyment, hope, and interest, were aggregated into composite scores by averaging their respective items. Agentic engagement was the independent variable, EFL achievement the dependent variable, and the composite emotion score—either negative or positive—the moderator. Interaction terms were generated and tested for significance using *p*-values.

Subsequently, multi-group analyses were performed using Amos to test both moderating and mediating effects for Grade One and Grade Four undergraduate student groups. First, for the moderation analysis, a multi-group path model was specified with agentic engagement as the independent variable, EFL achievement as the dependent variable, and composite emotion scores (positive or negative) as the moderator. The moderating effects of either positive emotions or negative emotions and their *p*-values for Grade One and Grade Four undergraduate student groups were estimated. Second, for the mediation analysis, a multi-group path model was examined within each cohort. The model specified agentic engagement as the independent variable, EFL achievement as the dependent variable, and composite emotion scores (positive or negative) as the mediator. The direct and indirect effects and their *p*-values for Grade One and Grade Four undergraduate student groups were estimated. Indirect effects were tested using bootstrapping with 5,000 resamples. An effect was considered statistically significant if the 95% bias-corrected bootstrap confidence interval did not include zero.

By focusing on discrete emotions and composite valence categories, and comparing across grade levels, the analysis plan captures both the specificity and breadth of emotional influences on engagement and achievement. The findings are expected to illuminate the complex interplay of these factors, offering a robust basis for theoretical advancement and practical application in Chinese EFL education.

## 4 Results

This section presents the findings from this study exploring the relationships among agentic engagement, academic emotions, and English achievement in a sample of 221 students from undergraduate grades one and four. These findings underscore the evolving role of emotions in educational contexts, particularly in how they interact with engagement to influence achievement in subjects like English.

### 4.1 Descriptive statistics

Based on the data collected from the FLL-AES (Guo and Li, 2018), the descriptive statistics in Table 1 offers insights into agentic engagement across different groups, including the whole sample, Grade One, and Grade Four undergraduate students. The reliability and validity of the FLL-AES based on the data of this study were satisfactory and consistent in different groups. The scale demonstrated excellent reliability for the whole sample (*α* = 0.92), for Grade One undergraduate students (*α* = 0.91), and for Grade Four undergraduate students (*α* = 0.93). The four-factor model fit the data fairly well in the whole sample, Grade One, and Grade Four undergraduate students. Confirmatory Factor Analysis indicated good model fit for the whole sample (CFI = 0.981, RMSEA = 0.056). Configural invariance was tested for by confirming the existence of an equivalent factor structure across groups. The model remained well-fitting (CFI = 0.974, RMSEA = 0.063), indicating that the basic factor model of the FLL-AES was equivalent across Grade One and Grade Four undergraduate students.

**Table 1:** Descriptive data of agentic engagement.

Descriptive statistics	Entire sample (*N* = 221)	Grade One (*N* = 111)	Grade Four (*N* = 110)
Mean	51.539	51.748	51.327
Standard deviation	8.513	9.395	7.557
Skewness	−0.083	−0.176	0.054
Kurtosis	0.940	0.861	0.738
Minimum value	18.00	18.00	28.00
Maximum value	70.00	70.00	70.00

Mean, standard deviation, skewness, kurtosis, minimum, and maximum values were adopted to describe the central tendencies, variability, and distributional characteristics of agentic engagement.

Agentic engagement for the entire sample of 221 participants showed a moderate overall level (Mean = 51.539) with moderate variability (SD = 8.513). The distribution was nearly symmetric (Skewness = −0.083, slight left tilt) and slightly more peaked than normal (Kurtosis = 0.940). Scores ranged widely from 18.00 to 70.00, indicating substantial diversity.

Comparing grade levels revealed subtle differences. Grade One undergraduate students exhibited a higher mean engagement (51.748) but greater variability (SD = 9.395), including the sample’s lowest score (18.00) and a more notable left-skew. This suggests younger students experience higher but more fluctuating engagement. Conversely, Grade Four undergraduate students showed a slightly lower mean (51.327) with tighter consistency (SD = 7.557) and a higher minimum score (28.00), alongside the flattest distribution. This pattern indicates greater behavioral stability as students mature.

The reliability and validity of the GAEQ (Ma, 2008) based on the data of this study were also satisfactory and consistent in different groups. Cronbach’s Alpha coefficients for the 10 subscales ranged from 0.652 to 0.892 for the whole sample, from 0.643 to 0.889 for Grade One undergraduate students, and from 0.668 to 0.901 Grade Four undergraduate students. Both the original four-factor model and the two-factor model (positive emotions and negative emotions) fit the data fairly well in the whole sample, Grade One, and Grade Four undergraduate students. Under the two-factor model, confirmatory factor analysis indicated good model fit for the whole sample (CFI = 0.931, RMSEA = 0.069). Configural invariance was established by fitting the unconstrained model with the same factor structure specified for both groups. The model fit was acceptable (CFI = 0.927, RMSEA = 0.073), indicating that the two-factor model of the GAEQ was equivalent across Grade One and Grade Four undergraduate students.

Based on the data collected from the GAEQ (Ma, 2008), the mean for each academic emotion was calculated by dividing the total score across the items of that emotion by the number of items measuring that specific emotion. Table 2 presents the mean scores for each emotion and emotion type.

**Table 2:** Descriptive data of academic emotions.

Emotion	Sum of items	Emotion type	Item score means for each emotion (Entire sample)		Item score means for each emotion (Grade One)		Item score means for each emotion (Grade Four)	
Anxiety	15	Negative emotions	3.163	2.932	3.182	2.926	3.144	2.938
Boredom	13		2.679		2.728		2.630	
Hopelessness	10		2.764		2.805		2.724	
Shame	7		3.131		3.081		3.182	
Anger	5		2.921		2.834		3.009	
Relief	10	Positive emotions	3.223	3.562	3.170	3.492	3.275	3.632
Pride	9		3.471		3.370		3.573	
Enjoyment	7		3.651		3.559		3.744	
Hope	7		3.893		3.810		3.977	
Interest	5		3.573		3.553		3.593	

Overall, the entire sample exhibits a clear distinction between positive and negative emotions, with emotions such as hope and enjoyment consistently demonstrating the highest mean scores, averaging 3.893 and 3.651 respectively, while boredom registers the lowest mean at 2.679, indicating a tendency toward more favorable affective states overall. In comparison, mid-range scores are observed for emotions like anxiety (3.163) and shame (3.131), suggesting a moderate prevalence of these feelings in the general population.

Delving into the grade-level comparisons, Grade Four undergraduate students report higher mean scores than Grade One undergraduate students across all positive emotions. However, Grade Four also exhibits a slight increase in some negative emotions like shame (3.182 vs. Grade One’s 3.081) and anger (3.009 vs. 2.834), while anxiety, boredom, and hopelessness decrease marginally from Grade One to Grade Four. This pattern underscores a general skew toward higher positive affect in the sample, with Grade Four undergraduate students displaying stronger overall emotional positivity. Negative emotions demonstrated relative stability between Grade One (Mean = 2.926) and Grade Four (Mean = 2.938), with minimal deviation from the whole-sample average (Mean = 2.932).

Analysis of first-grade (*N* = 111) and fourth-grade (*N* = 110) English exam scores reveals distinct performance patterns. Although the test items for each grade differ in content and difficulty, Rasch calibration and equating place the ability scores on a common scale.

First graders achieved a moderate mean score of 71.30 with high standard deviation (12.73), indicating substantial variability. Their distribution is left-skewed (skewness = −1.092), meaning more students scored above average, and near-normal kurtosis (0.966). Scores ranged from 28.00 to 90.00. Fourth graders showed a higher mean (82.42) and lower standard deviation (10.47), suggesting greater consistency. However, their distribution exhibited extreme left-skew (skewness = −3.053) and very high kurtosis (16.422), reflecting scores tightly clustered near the mean with a sharp peak and heavy tails. This indicates most performed well, but significant outliers exist, confirmed by the minimum (14.00) and maximum (95.00) scores.

While fourth graders demonstrate higher average achievement with less variability overall, their score distribution is dramatically more skewed and peaked compared to first graders. The pronounced differences in skewness and kurtosis highlight unique distribution characteristics for each grade.

### 4.2 Moderating and mediating roles of discrete academic emotions

The moderating effects of academic emotions on the relationship between agentic engagement and English achievement were examined among 221 students in grades one and four. As summarized in Table 3, the results revealed differential moderating roles of specific academic emotions.

**Table 3:** Moderating effects of academic emotions for the entire sample (*N* = 221).

Moderator	*β*
Anxiety	0.013
Boredom	0.017
Relief	−0.014
Hopelessness	0.029
Pride	−0.022
Shame	0.004
Enjoyment	−0.027
Hope	−0.022
Anger	0.035
Interest	−0.016

Dependent variable: English achievement.

Benjamini-Hochberg correction was considered.

In a sample of 221 students, hierarchical regression analysis was employed to examine the moderating effects of various academic emotions on the relationship between agentic engagement and English achievement, with *p*-values used to assess statistical significance. The results initially indicated that hopelessness demonstrated significant moderating effect (*β* = 0.029); however, this effect did not remain significant after applying the Benjamini-Hochberg correction for multiple comparisons. All other emotions showed nonsignificant moderation. Anger displayed the highest positive trend (*β* = 0.035), while negative emotions such as anxiety (*β* = 0.013) and boredom (*β* = 0.017) had small positive effects. Among positive emotions, pride (*β* = −0.022), hope (*β* = −0.022), and enjoyment (*β* = −0.027) exhibited weak negative trends, indicating a potential attenuation of the engagement–achievement link at higher levels of these emotions. Interest (*β* = −0.016) and shame (*β* = 0.004) contributed negligible variance, underscoring their minimal role as moderators in the model. Overall, while several emotions showed directional trends, none retained statistical significance following correction.

The mediating effects of academic emotions between agentic engagement and English achievement were analyzed in a sample of 221 students from grades one and four. The *p*-value thresholds (*p* < 0.01 or *p* < 0.05) displayed in Table 4 represent the desired significance level (*q*) applied in the Benjamini-Hochberg correction.

**Table 4:** Mediating effects of academic emotions for the entire sample (*N* = 221).

Mediator	Direct effect of agentic engagement on English achievement	Indirect effect through mediator
Anxiety	0.341**	0.054*
Boredom	0.347**	0.048
Relief	0.284*	0.111
Hopelessness	0.281*	0.115*
Pride	0.150	0.246*
Shame	0.406**	−0.010
Enjoyment	0.273*	0.123
Hope	0.266*	0.129
Anger	0.404**	−0.009
Interest	0.448**	−0.053

Dependent variable: English achievement.

**p < 0.01, *p < 0.05. Benjamini-Hochberg correction was considered.

A significant indirect effect (*p* < 0.05) was observed for pride while the direct effect between agentic engagement and English achievement was non-significant (*β* = 0.150), indicating complete mediation. The indirect effect through hopelessness was significant (*β* = 0.115*, *p* < 0.05) and the direct effect remained significant (*β* = 0.281*, *p* < 0.05), indicating partial mediation. While the direct effect of anxiety was strongly significant (*β* = 0.341**, *p* < 0.01), a marginally significant indirect effect (0.054*, *p* < 0.05) suggested weak complementary mediation.

Relief, enjoyment, and hope showed indirect effects of 0.111, 0.123, and 0.129, respectively, but these did not reach statistical significance. Emotions such as shame (indirect effect = −0.010), anger (indirect effect = −0.009), and interest (indirect effect = −0.053) demonstrated negligible or counterintuitive mediation pathways.

For most emotions, agentic engagement exerted strong direct effects on English achievement, overshadowing non-significant indirect pathways. This suggests that proactive learning behaviors primarily influence outcomes through mechanisms other than the tested emotional mediators (Tables 5–8).

**Table 5:** Multi-group moderation analyses with positive emotions as the moderator.

Moderator	Group	*β*	*p*
Positive emotions	Grade One	−0.002	0.733
	Grade Four	−0.001	0.901

**Table 6:** Multi-group moderation analyses with negative emotions as the moderator.

Moderator	Group	*β*	*p*
Negative emotions	Grade One	0.005	0.188
	Grade Four	0.004	0.200

**Table 7:** Multi-group mediation analyses with positive emotions as the mediator.

Mediator	Group	Direct effect of agentic engagement on English achievement	Indirect effect through mediator
Positive emotions	Grade One	0.514**	−0.002
	Grade Four	0.081	0.174*

Dependent variable: English achievement.

**p < 0.01, *p < 0.05.

**Table 8:** Multi-group mediation analyses with negative emotions as the mediator.

Mediator	Group	Direct effect of agentic engagement on English achievement	Indirect effect through mediator
Negative emotions	Grade One	0.495**	0.017
	Grade Four	0.189	0.066

Dependent variable: English achievement.

**p < 0.01.

### 4.3 Grade level comparisons of the moderating and mediating effects of academic emotions

By grouping negative emotions (anxiety, boredom, hopelessness, shame, anger) and positive emotions (relief, pride, enjoyment, hope, interest) into composite scores, moderation and mediation analyses were conducted at the grade level. Multi-group moderation and mediation analyses were conducted to examine the roles of positive and negative academic emotions in the relationship between agentic engagement and English achievement among Grade One (*N* = 111) and Grade Four (*N* = 110) students.

In the moderation analyses, neither positive nor negative emotions significantly moderated this relationship in either grade level. For Grade One undergraduate students, the moderating effect of positive emotions was non-significant (*β* = −0.002, *p* = 0.733), as was that of negative emotions (*β* = 0.005, *p* = 0.188). Similarly, for Grade Four undergraduate students, both moderators were non-significant—positive emotions: *β* = −0.001, *p* = 0.901; negative emotions: *β* = 0.004, *p* = 0.200—indicating that the strength of the association between agentic engagement and English achievement does not vary meaningfully as a function of emotional experience at either stage.

In contrast, the mediation analyses revealed distinct developmental patterns across grades. Among Grade One undergraduate students, agentic engagement had a strong and statistically significant direct effect on English achievement, both when positive emotions (*β* = 0.514, *p* < 0.01) and negative emotions (*β* = 0.495, *p* < 0.01) were included as mediators. However, the indirect effects through both emotional pathways were negligible: −0.002 for positive emotions and 0.017 for negative emotions. This indicates that while agentic engagement strongly predicts English achievement among first-year undergraduate students, this influence operates almost entirely through direct pathways rather than being transmitted via academic emotions.

A strikingly different pattern emerged for Grade Four undergraduate students. While the direct effect of agentic engagement on English achievement became non-significant when positive emotions were introduced as a mediator (*β* = 0.081, *p* > 0.05), the indirect effect through positive emotions was both large and statistically significant (0.174, *p* < 0.05). This satisfies the criteria for complete mediation, suggesting that the entire impact of agentic engagement on English achievement is accounted for by students’ positive emotional experiences. On the other hand, the indirect effect through negative emotions was not statistically significant (indirect effect = 0.066), indicating no reliable mediating role. Thus, negative emotions do not meaningfully transmit the influence of agentic engagement on achievement among fourth-year undergraduate students.

Overall, these findings highlight a developmental shift in the mechanisms linking student agency to academic outcomes. In Grade One, agentic engagement directly enhances English achievement without substantial involvement of emotional processes. By Grade Four, however, positive academic emotions become the central pathway through which agentic engagement influences learning success, underscoring their increasing importance in educational contexts as students grow older. Although neither positive nor negative emotions serve as moderators at either grade level, the emergence of complete mediation through positive emotions in later undergraduate years suggests that fostering emotionally supportive learning environments may be critical for maximizing the benefits of student agency in academic performance.

## 5 Discussion

The present study examined the relationship between agentic engagement and English as a foreign language (EFL) achievement among Chinese undergraduate students, with a particular focus on how academic emotions moderate and mediate this dynamic. The findings indicate a significant direct effect of agentic engagement on EFL achievement, especially pronounced among first-year undergraduate students, alongside a notable influence of emotions in shaping this relationship. By integrating theories from linguistics and psychology, this discussion interprets these results, situates them within the broader literature, and explores their implications within the Chinese educational context.

### 5.1 Agentic engagement and academic emotions of Chinese university EFL learners

The present study, conducted with 221 Chinese university EFL learners, provides valuable descriptive insights into students’ agentic engagement and academic emotions in foreign language learning. Agentic engagement, as measured by the FLL-AES (Guo and Li, 2018), revealed a moderate overall level (Mean = 51.54, SD = 8.51). The near-normal distribution (skewness = −0.08, kurtosis = 0.94) and wide score range (18–70) indicate considerable individual differences among Chinese undergraduates. The scale demonstrated strong psychometric properties in this cultural context, with excellent internal consistency (*α* = 0.92) and good structural validity (CFI = 0.981, RMSEA = 0.056), confirming its applicability to Chinese university students (Guo and Li, 2018; Reeve and Tseng, 2011).

Academic emotions assessed with the GAEQ (Ma, 2008) showed a clear predominance of positive over negative affect. Among positive emotions, hope (Mean = 3.89) and enjoyment (Mean = 3.65) emerged as the most frequently experienced, whereas boredom recorded the lowest mean (Mean = 2.68) among all emotions. Negative emotions clustered around moderate intensity (overall M ≈ 2.93), with anxiety (Mean = 3.16) and shame (Mean = 3.13) being the most salient. This positive emotional profile aligns with recent studies of Chinese EFL university students, who often report higher enjoyment and hope than boredom or hopelessness when learning English (e.g., Dewaele and Dewaele, 2017; Jiang and Dewaele, 2019).

These findings extend prior research by documenting both agentic engagement and emotional experiences within the same large sample of Chinese undergraduates, highlighting a generally favorable motivational-affective climate in tertiary EFL education. The moderate-to-high levels of agentic engagement and the dominance of positive emotions suggest that many Chinese university students actively contribute to their learning process and experience English classes as emotionally rewarding, which may partly explain sustained motivation despite known challenges in the Chinese EFL context (Wang and Li, 2022).

### 5.2 Agentic engagement as the predictor

Agentic engagement, as defined by Reeve and Tseng (2011), aligns closely with self-regulated learning theory from educational psychology, which posits that learners who actively manage their cognitive, motivational, and behavioral processes achieve better academic outcomes (Zimmerman, 2002). In the context of EFL learning, where acquiring a new language involves navigating linguistic complexity and cultural nuances, agentic engagement appears to be a critical driver of success (Jiang and Zhang, 2021; Zhong et al., 2025). The current study supports this, revealing that students who exhibit higher levels of engagement—particularly first-year undergraduate students adapting to university demands—demonstrate greater EFL achievement. For novice language learners, proactive engagement may serve as a mechanism to bridge their current abilities and potential development, fostering autonomy over time (Mercer, 2019).

The significant direct effect of agentic engagement on English achievement among first-year undergraduate students, contrasted with its non-significant effect among fourth-year undergraduate students, can be attributed to differences in developmental stages and academic experience. For first-year undergraduate students, who are typically novices in the university environment, these behaviors are critical for success in English achievement. Psychological research on self-regulated learning supports this, suggesting that novice learners depend heavily on active control of their learning processes to overcome initial challenges and achieve academic outcomes (Zimmerman, 2000). In contrast, fourth-year undergraduate students, having spent several years in the university system, are likely to have developed more refined academic habits and a deeper knowledge base in English. This accumulated expertise may reduce their dependence on direct agentic engagement, as they can draw on prior learning and more automatic strategies rather than effort-intensive proactive behaviors.

### 5.3 Roles of discrete academic emotions

Several patterns emerge regarding the role of academic emotions in the relationship between agentic engagement and English achievement. First, none of the academic emotions showed significant moderating effects, which suggests that emotional states did not change how strongly agentic engagement predicted achievement. This is consistent with the Control–Value Theory (Pekrun, 2006), which proposes that although emotions influence learning, they often exert direct or indirect effects rather than interacting with motivational behaviors. Prior research also indicates that academic emotions frequently operate as additive predictors of performance rather than moderators of other motivational processes (Goetz et al., 2006), which helps explain the uniformly weak moderating effects.

Pride stands out because it exhibited a full mediating effect, meaning agentic engagement influenced achievement entirely through increasing pride. Pride is a self-evaluative, achievement-related emotion rooted in perceived competence and successful self-initiated action (Tracy and Robins, 2007). Agentic engagement, which involves taking initiative, asking questions, and shaping learning conditions (Reeve, 2013), naturally enhances feelings of competence and accomplishment. Therefore, pride becomes the central emotional mechanism through which proactive engagement benefits achievement. Prior research indicates that pride is strongly associated with persistence, self-regulation, and performance (Pekrun and Linnenbrink-Garcia, 2012; Dewaele and Dewaele, 2020), which helps explain why the direct path from engagement to achievement became nonsignificant once pride was included. In other words, pride fully explains how engagement translates into improved English performance. This finding underscores the centrality of emotional pathways for advanced undergraduate students, where positive emotions like pride serve as catalytic mechanisms that translate proactive behaviors into academic success.

Both hopelessness and anxiety showed partial and positive mediating effects in the relationship between agentic engagement and English achievement. This indicates that higher levels of agentic engagement were associated with increases in these emotions, and these elevated emotional states in turn contributed positively—though modestly—to achievement. While counterintuitive, such positive mediation aligns with research suggesting that certain negative activating emotions can sometimes motivate learners to exert additional effort or regain control (Pekrun, 2006). Anxiety, for example, may prompt students to study more intensely to avoid failure, which can lead to performance gains under manageable levels of stress (MacIntyre and Gardner, 1994). Similarly, slight increases in hopelessness may reflect heightened awareness of academic challenges, encouraging learners to engage more deliberately with instructional support. Because agentic engagement directly enhances learners’ sense of responsibility and involvement (Reeve, 2013), its influence on these negative emotions may activate compensatory effort that contributes indirectly to achievement. However, the persistence of significant direct paths suggests that these emotions explain only part of the engagement–achievement link, consistent with the notion that emotional and behavioral factors jointly shape learning outcomes.

The effects of pride, hopelessness, and anxiety in this study illuminate the unique Chinese educational context, characterized by intense pressure and a deep cultural emphasis on academic achievement. Hopelessness, with its positive mediating roles, reflects the high stakes of education in China, where unmet expectations—often shaped by familial and societal demands—push students to work harder and improve, amplifying the impact of their engagement. Pride, as a complete mediator, highlights the cultural valorization of success, reinforcing students’ drive to excel as they internalize achievement as a source of personal and collective honor. Anxiety, while not so strong in effects, likely operates as an undercurrent in this high-pressure environment, potentially intensifying students’ emotional responses to both success and failure.

Other academic emotions do not demonstrate significant mediating effects in the relationship between agentic engagement and English achievement. This may be because their influence on the underlying cognitive and motivational processes is either limited or inconsistent (Shi and Wang, 2025). For instance, while negative emotions such as boredom and anger can detract from engagement, they appear to lack the direct and specific impact on achievement observed with hopelessness—possibly because they are less clearly tied to academic expectations. Similarly, positive emotions like enjoyment and hope may support learning in general but do not substantially alter the pathway from engagement to achievement, potentially due to their broader focus or relatively lower intensity in academic settings compared to the salient impact of pride.

### 5.4 Grade level differences concerning academic emotions

The findings from the General Academic Emotions Questionnaire (GAEQ; Ma, 2008) offer a window into the emotional landscape of first and fourth-grade undergraduate students, revealing distinct patterns in academic emotions. These results suggest a developmental trajectory where positive emotions strengthen and negative emotions shift as student’s progress through their early academic years. The data indicate that fourth-grade undergraduate students experience higher levels in all the positive emotions, compared to their first-grade peers, while negative emotions like anxiety, boredom, and hopelessness are more prominent among the younger cohort. This aligns with Pekrun’s Control-Value Theory of Achievement Emotions (2006), which posits that emotions stem from students’ appraisals of control and value in academic contexts. As students advance from first to fourth grade, they typically gain greater mastery over academic tasks, fostering a sense of competence that fuels positive emotions.

The differing roles of positive emotions as a mediator between agentic engagement and English achievement further highlight the impact of experience and emotional maturity. For fourth-year undergraduate students, positive emotions significantly mediate this relationship, whereas for first-year undergraduate students, this mediating effect is non-significant. This discrepancy can be explained through the lens of the broaden-and-build theory of positive emotions, which posits that positive emotions expand cognitive flexibility and resourcefulness, enhancing learning and performance (Fredrickson, 2001). Fourth-year undergraduate students, with greater emotional regulation skills honed over years of academic and personal growth, are better equipped to leverage positive emotions to sustain engagement and improve their English achievement (Burić and Frenzel, 2021). Conversely, first-year undergraduate students, still adjusting to the emotional turbulence of university life, may experience positive emotions that are less stable or less integrated into their learning processes. Research on academic emotions corroborates this, indicating that less experienced students struggle with emotion regulation, limiting their ability to harness emotions effectively for learning (Pekrun et al., 2002).

The grade-level difference in the mediating role of positive emotions can be theoretically grounded in developmental changes in self-regulation and emotion regulation during the transition from adolescence to emerging adulthood. First-year undergraduate students typically exhibit less mature executive functions and rely on rudimentary emotion regulation strategies (Steinberg et al., 2008; Zimmermann and Iwanski, 2014), making it difficult to effectively channel positive academic emotions into sustained agentic engagement; thus, the effect on achievement remains direct rather than mediated. By the fourth year, significant maturation in self-regulatory abilities, including improved cognitive reappraisal and future-oriented thinking (McRae et al., 2012), together with greater identity consolidation (Arnett, 2000), enables senior students to more effectively harness positive emotions to “broaden and build” resources (Fredrickson, 2001), thereby transforming these emotions into a robust mediator between agentic engagement and English achievement.

The cultural context of this study—China’s examination-oriented educational system—further enriches the interpretation of these results. The prevalence of negative emotions among first-year undergraduate students likely reflects the high-stakes nature of academic transitions within this system. Yet, the observed decrease in negative emotions and increase in positive emotions by the fourth year suggest an emotional adaptation as students gain proficiency and confidence. This aligns with sociocultural views of EFL learning as a socially mediated process, where emotional and behavioral adjustments occur in response to contextual demands (Lantolf and Thorne, 2006).

### 5.5 Toward an integrated conceptual model

This study integrates Control-Value Theory (Pekrun, 2006), self-regulated learning (SRL; Zimmerman, 2002), and the broaden-and-build theory (Fredrickson, 2001) into a cohesive framework explaining how agentic engagement influences EFL achievement through emotional mechanisms.

According to this model, self-regulated learning theory (Zimmerman, 2000, 2002) conceptualizes agentic engagement (Reeve and Tseng, 2011) as proactive self-initiation that directly enhances achievement, especially among first-year undergraduate students. Control-value theory (Pekrun, 2006) elucidates emotion generation: higher agentic engagement increases perceived control and value, eliciting strong pride and moderate anxiety/hopelessness. In the Chinese high-stakes context, pride fully mediates the engagement–achievement link overall and dominates among fourth-year undergraduate students, while anxiety and hopelessness exert partial positive mediation by spurring compensatory effort.

The broaden-and-build theory (Fredrickson, 2001) explains developmental differences: positive emotions broaden cognition and build enduring resources, but this process strengthens with experience and regulatory maturity. Consequently, fourth-year undergraduate students rely predominantly on emotion-mediated paths (especially pride), whereas first-year undergraduate students exhibit stronger direct engagement–achievement effects and weaker positive-emotion mediation.

Together, these theories form a dynamic, context-sensitive model in which agentic behaviors and academic emotions interact differentially across stages of academic development. This integrated perspective underscores the need to view motivation, emotion, and learning as interdependent processes, particularly within culturally specific, high-stakes educational environments like China’s EFL context.

## 6 Conclusion

This study explored the intricate interplay between agentic engagement, academic emotions, and EFL achievement among Chinese undergraduate students, addressing three pivotal research questions concerning the roles of discrete, negative, and positive emotions across different grade levels. The investigation revealed that pride emerged as a substantial mediator, particularly among advanced learners. Hopelessness and anxiety showed partial mediation while other emotions showed limited or non-significant effects. When examining grade-specific dynamics, the findings indicated that for first-year undergraduate students, agentic engagement exerted a direct influence on EFL achievement, with neither positive nor negative emotions playing a significant moderating or mediating role. In contrast, for fourth-year undergraduate students, positive emotions fully mediated the relationship between agentic engagement and EFL achievement, whereas negative emotions exhibited no significant mediating effect. These results underscore the varying emotional influences on engagement and achievement as student’s progress through their academic journey.

Despite these insights, an important limitation of the study lies in that the sample was confined to English majors from a single university in China, potentially limiting the generalizability of the findings to other disciplines, institutions, or cultural contexts. However, the decision to focus on one institution was primarily driven by practical and methodological considerations, including access to a homogeneous and well-defined population and the need to maintain consistency in curriculum, instruction, and assessment across participants. Similarly, the deliberate selection of English majors strengthens the study’s theoretical relevance, as these students are deeply immersed in EFL learning, making them ideal for investigating the dynamics between agency, emotions, and achievement in a high-involvement context.

Theoretically, this research enriches the understanding of how discrete academic emotions interact with agentic engagement to shape EFL achievement, particularly within a non-Western educational framework. It extends Pekrun’s (2006) control-value theory by illustrating how emotions like hopelessness and pride modulate engagement’s effectiveness in a language learning context, and complements Zimmerman’s (2002) self-regulated learning theory by highlighting the developmental shifts in these processes. The study’s focus on Chinese learners adds a culturally contingent dimension to these frameworks, emphasizing the need for stage-specific models that account for emotional and behavioral adaptations across university years.

Practically, the findings offer valuable implications for educators in EFL settings. For first-year undergraduate students, fostering agentic engagement through strategies such as goal-setting and feedback-seeking appears to directly enhance achievement, suggesting that early interventions should prioritize building proactive learning habits. For fourth-year undergraduate students, cultivating positive emotions like pride and enjoyment could be instrumental in sustaining engagement and boosting performance, particularly in China’s high-stakes, examination-oriented system where emotional experiences are intensified. These tailored approaches can help mitigate negative affect and leverage positive emotional states to optimize EFL success.

In sum, this study bridges critical gaps in the literature by elucidating the roles of academic emotions in the relationship between agentic engagement and EFL achievement, offering a nuanced perspective on their interplay across different stages of university education. Future research should adopt longitudinal designs and broader samples to further validate and expand these findings, ultimately informing more effective pedagogical strategies for supporting Chinese undergraduate students in their language learning endeavors.

## Data Availability

The original contributions presented in the study are included in the article/Supplementary material, further inquiries can be directed to the corresponding author.
