# Supplementary material for: Investigating the roles of academic emotions in the relationship between agentic engagement and EFL achievement among Chinese undergraduate students
Source: Front Psychol. 2025 Dec 17;16:1661196. doi: 10.3389/fpsyg.2025.1661196 (PMC12753975; doi:10.3389/fpsyg.2025.1661196)
Supplement: Supplementary file 1 [file Supplementary_file_1.docx]

Appendix A: English translation of Foreign Language Learning Agentic Engagement Scale (FLL-AES) Guo et al. (2018)

1. I listen attentively during English classes.

1=Completely disagree 2=Slightly disagree 3=Neutral 4=Somewhat agree 5=Completely agree

1. I let my English teacher know about my learning interests or needs.

1=Completely disagree 2=Slightly disagree 3=Neutral 4=Somewhat agree 5=Completely agree

1. I offer suggestions or feedback to the teacher on how to improve English teaching effectiveness.

1=Completely disagree 2=Slightly disagree 3=Neutral 4=Somewhat agree 5=Completely agree

1. I provide the teacher with some English materials and relevant information.

1=Completely disagree 2=Slightly disagree 3=Neutral 4=Somewhat agree 5=Completely agree

1. I actively participate in classroom activities organized by the English teacher.

1=Completely disagree 2=Slightly disagree 3=Neutral 4=Somewhat agree 5=Completely agree

1. I preview the content that the English teacher will cover in class in advance.

1=Completely disagree 2=Slightly disagree 3=Neutral 4=Somewhat agree 5=Completely agree

1. I actively complete the English learning tasks assigned by the teacher.

1=Completely disagree 2=Slightly disagree 3=Neutral 4=Somewhat agree 5=Completely agree

1. I encourage my classmates to learn English well.

1=Completely disagree 2=Slightly disagree 3=Neutral 4=Somewhat agree 5=Completely agree

1. I share relevant English learning materials or information with my classmates.

1=Completely disagree 2=Slightly disagree 3=Neutral 4=Somewhat agree 5=Completely agree

1. I help my classmates overcome difficulties in learning English.

1=Completely disagree 2=Slightly disagree 3=Neutral 4=Somewhat agree 5=Completely agree

1. I don't put much effort into learning English.

1=Completely disagree 2=Slightly disagree 3=Neutral 4=Somewhat agree 5=Completely agree

1. I cultivate a positive attitude toward learning English.

1=Completely disagree 2=Slightly disagree 3=Neutral 4=Somewhat agree 5=Completely agree

1. I actively confront and resolve difficulties in learning English.

1=Completely disagree 2=Slightly disagree 3=Neutral 4=Somewhat agree 5=Completely agree

1. I improve my English proficiency through various methods.

1=Completely disagree 2=Slightly disagree 3=Neutral 4=Somewhat agree 5=Completely agree

Appendix B: English translation of General Academic Emotion Questionnaire for College Students (GAEQ) Ma (2008)

1. I always feel very nervous before exams.
   1=Completely disagree 2=Slightly disagree 3=Neutral 4=Somewhat agree 5=Completely agree
2. I feel sleepy as soon as I start studying.
   1=Completely disagree 2=Slightly disagree 3=Neutral 4=Somewhat agree 5=Completely agree
3. I can always study with peace of mind.
   1=Completely disagree 2=Slightly disagree 3=Neutral 4=Somewhat agree 5=Completely agree
4. I feel helpless as some courses become increasingly difficult to learn.
   1=Completely disagree 2=Slightly disagree 3=Neutral 4=Somewhat agree 5=Completely agree
5. Learning makes me feel fulfilled, and I'm proud of it.
   1=Completely disagree 2=Slightly disagree 3=Neutral 4=Somewhat agree 5=Completely agree
6. I feel guilty for not studying well towards my family and teachers.
   1=Completely disagree 2=Slightly disagree 3=Neutral 4=Somewhat agree 5=Completely agree
7. Learning makes me happy.
   1=Completely disagree 2=Slightly disagree 3=Neutral 4=Somewhat agree 5=Completely agree
8. I believe my studies will improve.
   1=Completely disagree 2=Slightly disagree 3=Neutral 4=Somewhat agree 5=Completely agree
9. I get angry when others say I study worse than them.
   1=Completely disagree 2=Slightly disagree 3=Neutral 4=Somewhat agree 5=Completely agree
10. I find learning interesting.
    1=Completely disagree 2=Slightly disagree 3=Neutral 4=Somewhat agree 5=Completely agree
11. Some courses, like basic subjects with too much memorization, make me anxious as I can't study them well.
    1=Completely disagree 2=Slightly disagree 3=Neutral 4=Somewhat agree 5=Completely agree
12. I'm easily restless when studying.
    1=Completely disagree 2=Slightly disagree 3=Neutral 4=Somewhat agree 5=Completely agree
13. I can complete learning tasks easily.
    1=Completely disagree 2=Slightly disagree 3=Neutral 4=Somewhat agree 5=Completely agree
14. I lack confidence in my studies.
    1=Completely disagree 2=Slightly disagree 3=Neutral 4=Somewhat agree 5=Completely agree
15. I don't think I'm worse than others in learning.
    1=Completely disagree 2=Slightly disagree 3=Neutral 4=Somewhat agree 5=Completely agree
16. I feel guilty towards myself when I could have done better in some courses but didn't.
    1=Completely disagree 2=Slightly disagree 3=Neutral 4=Somewhat agree 5=Completely agree
17. Sometimes I feel happy after completing an assignment.
    1=Completely disagree 2=Slightly disagree 3=Neutral 4=Somewhat agree 5=Completely agree
18. I hope I can study better.
    1=Completely disagree 2=Slightly disagree 3=Neutral 4=Somewhat agree 5=Completely agree
19. I get angry when frequently encountering setbacks in learning.
    1=Completely disagree 2=Slightly disagree 3=Neutral 4=Somewhat agree 5=Completely agree
20. I can always concentrate when studying.
    1=Completely disagree 2=Slightly disagree 3=Neutral 4=Somewhat agree 5=Completely agree
21. I'm worried when my grades don't improve.
    1=Completely disagree 2=Slightly disagree 3=Neutral 4=Somewhat agree 5=Completely agree
22. I'm easily irritated when studying.
    1=Completely disagree 2=Slightly disagree 3=Neutral 4=Somewhat agree 5=Completely agree
23. I have a calm mind when studying.
    1=Completely disagree 2=Slightly disagree 3=Neutral 4=Somewhat agree 5=Completely agree
24. I feel depressed when studying.
    1=Completely disagree 2=Slightly disagree 3=Neutral 4=Somewhat agree 5=Completely agree
25. I feel proud when I can easily complete learning tasks.
    1=Completely disagree 2=Slightly disagree 3=Neutral 4=Somewhat agree 5=Completely agree
26. I feel guilty for not getting into a good university.
    1=Completely disagree 2=Slightly disagree 3=Neutral 4=Somewhat agree 5=Completely agree
27. I'm happy when studying.
    1=Completely disagree 2=Slightly disagree 3=Neutral 4=Somewhat agree 5=Completely agree
28. I hope to achieve my learning goals.
    1=Completely disagree 2=Slightly disagree 3=Neutral 4=Somewhat agree 5=Completely agree
29. I get annoyed when I can't understand a lesson.
    1=Completely disagree 2=Slightly disagree 3=Neutral 4=Somewhat agree 5=Completely agree
30. I can always focus on studying.
    1=Completely disagree 2=Slightly disagree 3=Neutral 4=Somewhat agree 5=Completely agree
31. Learning makes me miserable.
    1=Completely disagree 2=Slightly disagree 3=Neutral 4=Somewhat agree 5=Completely agree
32. I hate learning.
    1=Completely disagree 2=Slightly disagree 3=Neutral 4=Somewhat agree 5=Completely agree
33. I can face exams with ease.
    1=Completely disagree 2=Slightly disagree 3=Neutral 4=Somewhat agree 5=Completely agree
34. I feel helpless about my studies.
    1=Completely disagree 2=Slightly disagree 3=Neutral 4=Somewhat agree 5=Completely agree
35. I'm satisfied with my academic performance.
    1=Completely disagree 2=Slightly disagree 3=Neutral 4=Somewhat agree 5=Completely agree
36. Sometimes I feel ashamed when my grades are worse than others'.
    1=Completely disagree 2=Slightly disagree 3=Neutral 4=Somewhat agree 5=Completely agree
37. I feel happy when I can solve all the problems in my homework correctly.
    1=Completely disagree 2=Slightly disagree 3=Neutral 4=Somewhat agree 5=Completely agree
38. I think learning is useful.
    1=Completely disagree 2=Slightly disagree 3=Neutral 4=Somewhat agree 5=Completely agree
39. I get angry when disturbed by others while studying.
    1=Completely disagree 2=Slightly disagree 3=Neutral 4=Somewhat agree 5=Completely agree
40. I'm curious about every new aspect of learning.
    1=Completely disagree 2=Slightly disagree 3=Neutral 4=Somewhat agree 5=Completely agree
41. I feel anxious when I can't study language subjects like college English well.
    1=Completely disagree 2=Slightly disagree 3=Neutral 4=Somewhat agree 5=Completely agree
42. I find learning boring.
    1=Completely disagree 2=Slightly disagree 3=Neutral 4=Somewhat agree 5=Completely agree
43. I can take my grades calmly.
    1=Completely disagree 2=Slightly disagree 3=Neutral 4=Somewhat agree 5=Completely agree
44. I feel powerless in my studies.
    1=Completely disagree 2=Slightly disagree 3=Neutral 4=Somewhat agree 5=Completely agree
45. I feel proud as learning has enriched my knowledge and skills.
    1=Completely disagree 2=Slightly disagree 3=Neutral 4=Somewhat agree 5=Completely agree
46. Sometimes I feel ashamed in front of others when my grades are poor.
    1=Completely disagree 2=Slightly disagree 3=Neutral 4=Somewhat agree 5=Completely agree
47. I always study happily.
    1=Completely disagree 2=Slightly disagree 3=Neutral 4=Somewhat agree 5=Completely agree
48. I'm confident in my studies.
    1=Completely disagree 2=Slightly disagree 3=Neutral 4=Somewhat agree 5=Completely agree
49. I get angry when the teacher doesn't ask me questions.
    1=Completely disagree 2=Slightly disagree 3=Neutral 4=Somewhat agree 5=Completely agree
50. I have a desire to explore every new field of learning.
    1=Completely disagree 2=Slightly disagree 3=Neutral 4=Somewhat agree 5=Completely agree
51. Sometimes I feel miserable when my grades are poor.
    1=Completely disagree 2=Slightly disagree 3=Neutral 4=Somewhat agree 5=Completely agree
52. I often feel dizzy and confused when studying.
    1=Completely disagree 2=Slightly disagree 3=Neutral 4=Somewhat agree 5=Completely agree
53. I feel relaxed when doing my homework.
    1=Completely disagree 2=Slightly disagree 3=Neutral 4=Somewhat agree 5=Completely agree
54. I often encounter setbacks in my studies.
    1=Completely disagree 2=Slightly disagree 3=Neutral 4=Somewhat agree 5=Completely agree
55. I often receive praise and commendation from others for my studies.
    1=Completely disagree 2=Slightly disagree 3=Neutral 4=Somewhat agree 5=Completely agree
56. Sometimes I feel sad when my grades are worse than others'.
    1=Completely disagree 2=Slightly disagree 3=Neutral 4=Somewhat agree 5=Completely agree
57. I have a high enthusiasm for learning.
    1=Completely disagree 2=Slightly disagree 3=Neutral 4=Somewhat agree 5=Completely agree
58. I have hope for my future.
    1=Completely disagree 2=Slightly disagree 3=Neutral 4=Somewhat agree 5=Completely agree
59. I feel distressed when I don't understand the learning content.
    1=Completely disagree 2=Slightly disagree 3=Neutral 4=Somewhat agree 5=Completely agree
60. Heavy learning tasks make me irritable.
    1=Completely disagree 2=Slightly disagree 3=Neutral 4=Somewhat agree 5=Completely agree
61. I feel relaxed as my grades have been stable.
    1=Completely disagree 2=Slightly disagree 3=Neutral 4=Somewhat agree 5=Completely agree
62. Sometimes studying makes me feel dejected.
    1=Completely disagree 2=Slightly disagree 3=Neutral 4=Somewhat agree 5=Completely agree
63. I often feel smart in my studies.
    1=Completely disagree 2=Slightly disagree 3=Neutral 4=Somewhat agree 5=Completely agree
64. Sometimes I feel embarrassed when I can't solve problems that should be within my professional knowledge.
    1=Completely disagree 2=Slightly disagree 3=Neutral 4=Somewhat agree 5=Completely agree
65. Sometimes studying brings me surprises.
    1=Completely disagree 2=Slightly disagree 3=Neutral 4=Somewhat agree 5=Completely agree
66. Others' encouragement fills me with hope for learning.
    1=Completely disagree 2=Slightly disagree 3=Neutral 4=Somewhat agree 5=Completely agree
67. I feel anxious about learning.
    1=Completely disagree 2=Slightly disagree 3=Neutral 4=Somewhat agree 5=Completely agree
68. I think learning is difficult.
    1=Completely disagree 2=Slightly disagree 3=Neutral 4=Somewhat agree 5=Completely agree
69. I can handle learning with ease.
    1=Completely disagree 2=Slightly disagree 3=Neutral 4=Somewhat agree 5=Completely agree
70. I'm pessimistic about my future.
    1=Completely disagree 2=Slightly disagree 3=Neutral 4=Somewhat agree 5=Completely agree
71. I make faster progress than others in learning.
    1=Completely disagree 2=Slightly disagree 3=Neutral 4=Somewhat agree 5=Completely agree
72. I'm confused why I can't study well.
    1=Completely disagree 2=Slightly disagree 3=Neutral 4=Somewhat agree 5=Completely agree
73. Learning is a burden for me.
    1=Completely disagree 2=Slightly disagree 3=Neutral 4=Somewhat agree 5=Completely agree
74. I'm satisfied with my current learning situation.
    1=Completely disagree 2=Slightly disagree 3=Neutral 4=Somewhat agree 5=Completely agree
75. Sometimes I think I can't study well because I'm stupid.
    1=Completely disagree 2=Slightly disagree 3=Neutral 4=Somewhat agree 5=Completely agree
76. I feel proud when I achieve good grades.
    1=Completely disagree 2=Slightly disagree 3=Neutral 4=Somewhat agree 5=Completely agree
77. I don't know what to do as professional courses are too difficult to learn.
    1=Completely disagree 2=Slightly disagree 3=Neutral 4=Somewhat agree 5=Completely agree
78. I have no interest in learning.
    1=Completely disagree 2=Slightly disagree 3=Neutral 4=Somewhat agree 5=Completely agree
79. I'm generally relaxed in class.
    1=Completely disagree 2=Slightly disagree 3=Neutral 4=Somewhat agree 5=Completely agree
80. Despite my efforts, my grades don't improve.
    1=Completely disagree 2=Slightly disagree 3=Neutral 4=Somewhat agree 5=Completely agree
81. I worry about not studying well.
    1=Completely disagree 2=Slightly disagree 3=Neutral 4=Somewhat agree 5=Completely agree
82. I'm tired of learning.
    1=Completely disagree 2=Slightly disagree 3=Neutral 4=Somewhat agree 5=Completely agree
83. I worry about not studying well.
    1=Completely disagree 2=Slightly disagree 3=Neutral 4=Somewhat agree 5=Completely agree
84. I hate learning.
    1=Completely disagree 2=Slightly disagree 3=Neutral 4=Somewhat agree 5=Completely agree
85. Some learning content, like math and methodological courses, is too boring, making me anxious as I can't study them well.
    1=Completely disagree 2=Slightly disagree 3=Neutral 4=Somewhat agree 5=Completely agree
86. I think learning is a hard task.
    1=Completely disagree 2=Slightly disagree 3=Neutral 4=Somewhat agree 5=Completely agree
87. I worry about having worse grades than my classmates.
    1=Completely disagree 2=Slightly disagree 3=Neutral 4=Somewhat agree 5=Completely agree
88. Despite my efforts, I still can't study well and don't know what to do.
    1=Completely disagree 2=Slightly disagree 3=Neutral 4=Somewhat agree 5=Completely agree

Distribution of items in GAEQ subscales:

| Subscale | Items |
| --- | --- |
| Anxiety | 1, 11,21, 31, 41, 51, 59, 67, 72, 77, 81, 83, 85, 87, 88 |
| Boredom | 2, 12, 22, 32, 42, 52, 60, 68, 73, 78, 82, 84, 86 |
| Relief | 3, 13, 23, 33, 43, 53, 61, 69, 74, 79 |
| Hopelessness | 4, 14, 24, 34, 44, 54, 62, 70, 75, 80 |
| Pride | 5, 15, 25, 35, 45, 55, 63, 71, 76 |
| Shame | 6, 16, 26, 36, 46, 56, 64 |
| Enjoyment | 7, 17, 27, 37, 47, 57, 65 |
| Hope | 8, 18, 28, 38, 48, 58, 66 |
| Anger | 9, 19, 29, 39, 49 |
| Interest | 10, 20, 30, 40, 50 |
